# Supplementary material for: Construction and validation of m6A-related diagnostic model for psoriasis
Source: PeerJ. 2024 Feb 29;12:e17027. doi: 10.7717/peerj.17027 (PMC10909359; doi:10.7717/peerj.17027)
Supplement: Supplemental Information 2 [file peerj-12-17027-s002.docx]

**Table S1 Clincal information of patients involved in this study**

| **Variables** |  |
| --- | --- |
| No. of patients | 6 |
| **Age (years, mean)** | 47.75±14.01 |
| **Sex (%)**  Male  female | 3 (50%)  3 (50%) |
| **Body Surface Area (%)**  ＜3  3-10  ＞10 | 0  2 (33.3%)  4 (66.7%) |
| **Family history (%)**  Yes  No | 1 (16.7%)  5 (83.3%) |

**Table S2 Sequrences of primers for RT-qPCR**

| m6A genes | Forward ( 5’-3’) | Reverse ( 5’-3’) |
| --- | --- | --- |
| h-METTL3 | TTGTCTCCAACCTTCCGTAGT | CCAGATCAGAGAGGTGGTGTAG |
| h-YTHDC2 | CAACTCCTAGTAATGAACGGAAGC | TTAAATACTCCTCCTAGTCCAGC |
| h-IGF2BP2 | AGTGGAATTGCATGGGAAAATCA | CAACGGCGGTTTCTGTGTC |
| h-GAPDH | CTGGGCTACACTGAGCACC | AAGTGGTCGTTGAGGGCAATG |
| m-METTL3 | GGACTCTGGGCACTTGGATT | GCACGGGACTATCACTACGG |
| m-YTHDC2 | ATGGATCAGAAACAGCTCATGC | ATTGTTGAGTCGCCCGCTTG |
| m-IGF2BP2 | GGCCTTTGAGAACGACATGC | TGCACTGAGGTTTAGCCCTG |
| m-GAPDH | GGTTGTCCTGCGACTTCA | TCCTCCAGGGTTTCTTACTCC |
